# Submicroscopic infection of placenta by *Plasmodium* produces Th1/Th2 cytokine imbalance, inflammation and hypoxia in women from north-west Colombia

**DOI:** 10.1186/1475-2875-13-122

**Published:** 2014-03-27

**Authors:** Olga M Agudelo, Beatriz H Aristizabal, Stephanie K Yanow, Eliana Arango, Jaime Carmona-Fonseca, Amanda Maestre

**Affiliations:** 1 Grupo “Salud y Comunidad-César Uribe Piedrahita”, Universidad de Antioquia, Medellin, Colombia; 2 Hospital Pablo Tobón Uribe, Laboratorio de Biología Molecular, Medellín, Colombia; 3 School of Public Health, University of Alberta; Provincial Laboratory for Public Health, Edmonton, Canada

**Keywords:** Submicroscopic, Placental malaria, *Plasmodium*, Cytokine, Inflammation, Hypoxia

## Abstract

**Background:**

A large-scale study was set up in order to study the epidemiology, clinical aspects, and immunopathology of gestational and placental malaria in north-west Colombia. In this region, recent reports using a qPCR technique, confirmed frequencies of infection, by *Plasmodium falciparum* or *Plasmodium vivax,* up to 45%. Given the high rates of infection observed both in mother and placenta, a first exploratory study was proposed in order to characterize the effect on the inflammation status, tissue damage and hypoxia in *Plasmodium spp.* infected placentas.

**Methods:**

A descriptive, prospective, cross-sectional design was applied to pregnant women with (PM+) and without (PM-) placental malaria. Messenger RNA expression of Fas, FasL; COX-1, COX-2, HIF, VEGF, and the cytokines IL-2, IL-4, IL-10, IFN-γ and TNF, were measured in peripheral and placental blood using a quantitative PCR. The percentage of apoptotic cells was determined with a TUNEL assay.

**Results:**

In total 50 placentas were studied: 25 were positive for submicroscopic infection and 25 were negative for *Plasmodium* infection. Expression of IL-4 and IL-10 was observed high in placental tissue of PM+, while IL-2 was high in peripheral blood of the same group. Expression of TNF and IFNγ in peripheral blood of the PM + group was high. Similarly, the apoptotic index and Fas expression were significantly high in PM+. However, FasL expression was observed low in PM + compared to PM-. Inflammation markers (HIF, VEGF) and hypoxia markers (COX-1, COX-2) were high in the PM + group.

**Conclusion:**

During placental malaria expression of some pro-inflammatory cytokines is up-regulated and markers of hypoxia and tissue damage are increased in cases of submicroscopic infection.

## Background

Globally, an estimated 125 million pregnancies are at risk of malaria each year [1] and the disease causes substantial maternal and infant morbidity/mortality [2]. Both *Plasmodium falciparum* and *Plasmodium vivax* can cause adverse pregnancy outcomes, including maternal anaemia and low birth weight, albeit different mechanisms appear to be involved depending on the infecting species [3]. In the case of *P. falciparum*, adherence of infected erythrocytes to chondroitin sulfate A (CSA), present on the trophoblast surface, is crucial in the pathophysiology of the disease [4], since parasite sequestration results in inflammatory responses that can be harmful to both the mother and the foetus [5].

Women inhabiting areas with different degrees of endemicity experience variable exposure to infection and this affects the course of disease [6]. In areas of high stable transmission, as in most sub-Saharan regions, women have significant clinical immunity before pregnancy, and placental malaria is often asymptomatic, but associated with severe maternal anaemia and foetal growth retardation. In these regions, placental malaria is most frequent and severe in first time mothers, and women develop specific immunity against placental forms of infected erythrocytes over successive pregnancies. On the other hand, in areas of low unstable transmission, malaria is symptomatic in women of all parities and is associated with high rates of foetal loss and maternal death [7]. In such regions, increased susceptibility of primigravidae, compared to multigravidae, has been rarely documented [8].

Recent extensive reviews have focused on the pathogenesis and immunity of gestational and placental malaria caused by *P. falciparum*, specifically the molecular aspects of these infections, the associated damage to the placenta, and the consequences on the mother, the placenta and the foetus [3]. A significant role for increased cytokines (TNF, IFN-γ and IL-10) and apoptosis in trophoblast damage, low birth weight and preterm delivery during placental malaria, has been observed [3]. Some studies concluded that complications during pregnancy and foetal death observed in *P. falciparum* placental malaria might be determined by changes in the regulation of apoptosis, via Fas /FasL [9], and increased Th1 immune responses in the syncytiotrophoblast [10,11]. In addition, few reports identified a relationship between hypoxia and placental malaria [12,13], although an association between hypoxia and intrauterine growth retardation was observed in preeclampsia [14]. As for *P. vivax* infection during pregnancy, these phenomena remain to be studied.

On a routine basis, detection of parasites in placenta requires examination of histological sections of fixed placental tissue or microscopy of placental blood [15]. However, application of highly sensitive molecular techniques confirmed that the frequency of low level (submicroscopic) infections is higher than expected in all endemic regions [16,17]. Sensitive molecular methods typically diagnose approximately twice as many infections as microscopy. Different authors have suggested that even such low parasitaemia infections can result in pregnancy complications such as maternal anaemia and low birth weight [18]. However, the epidemiological characteristics and the consequences associated with submicroscopic plasmodial infection in pregnant women have been poorly evaluated [19,20].

Most knowledge gathered about pregnancy-associated malaria resulted from studies in areas of Africa where *P. falciparum* is highly endemic. In Colombia, where *P. vivax* represents 60-70% of the total reported cases, malaria in pregnancy constitutes a public health problem [15,21] with a high frequency of submicroscopic infections [16,15]. During the past 5 years, several reports contributed to the definition and understanding of the problem of gestational malaria in north-west Colombia [15,16,22]. To further recognize the effect of malaria infection on the pathophysiology of placental damage and to define the significance of low level infections in this context, a study was conducted to explore apoptosis, inflammation, and hypoxia in placental tissue, and to measure cytokines in maternal and placental blood, in relation to the presence of submicroscopic placental infection.

## Methods

### Study site

Women were recruited from January 2007 to April 2011, at the hospital obstetric facilities in the municipalities of Monteria (08°45′N, 75°53′W), and Puerto Libertador (07°53′35′ N, 75°40″16″ W) of the Uraba-Sinu-San Jorge-Bajo Cauca region of Colombia. This region has an estimated area of 43,506 km2, a population of 2.5 million at risk of malaria, with a mean annual parasite index of 35.8 cases/1,000 inhabitants [23]. The region has a low and stable malaria transmission intensity, with no marked fluctuations in the number of malaria cases during the year. The recorded prevalence of gestational malaria in the region was 9.1% by thick smear and 14.0% by PCR, while placental malaria was observed in 3.3% and 16.5%, respectively [24].

### Study design and sample selection

The study was part of a larger project aimed at exploring the epidemiology, clinical aspects and immunopathology of gestational and placental malaria in north-west Colombia, from which partial results have been reported elsewhere [21,24]. A total 2,000 pregnant women were recruited in a sequential fashion for the main study. Clinical and epidemiological surveys were applied to all those women. Based on the availability and quality of material collected from peripheral blood and placental tissue, a subset of subjects was selected from the records of the 2,000 women recruited in the main project, to explore the effect of malaria infection on the pathophysiology of placental damage. Regardless of the time of the year or the year of collection, a total 50 subjects, non-matched, were studied: 25 with confirmed placental infection and 25 without *Plasmodium spp.* infection. The status of infection was defined by thick smear and real-time PCR (qPCR) of placental blood. Positive infected units were those with a negative diagnosis by microscopy and a positive qPCR result; negative units were negative by both tests.

### Inclusion and exclusion criteria

Inclusion criteria were voluntary acceptance to participate in the study, signature of the informed consent, permanent residency (>1 year) in the malaria endemic region, negative history of pre-eclampsia and negative HIV and TORCH tests. The exclusion criterion was withdrawal of consent.

### Data and specimen collection

After enrolment, a questionnaire was completed which recorded data including age, number of pregnancies, number of malaria episodes during the ongoing pregnancy (based on antenatal records) and antimalarial treatment administered. In addition, labour/delivery and infant outcomes data, as well as maternal haemoglobin value, were obtained from the delivery chart. Placentas were processed immediately after delivery and maternal peripheral blood was obtained within 24 hours. After cleaning with saline, two ~1 cm^3^ sections of placenta were removed from the maternal side of the organ and blood was collected by aspiration into a tube containing EDTA. From mothers, 3 ml of peripheral blood was obtained and collected into EDTA containing vials. Thick smears were prepared from placental and mother’s blood and blood spots (approximately 100 μl of blood) were placed onto Whatman 3 mm filter paper for molecular diagnosis. One tissue fragment was preserved in RNA later® before processing for expression of markers of apoptosis, hypoxia and inflammation, and the remaining tissue fragment was fixed with 10% buffered formalin (pH 7.0-7.4), before embedding in paraffin, for the TUNEL assay.

### Diagnosis of infection by *Plasmodium*

*Plasmodium* infection was evaluated in mother’s peripheral and placental blood by microscopy of Field-stained thick films and qPCR. An experienced microscopist based at the field laboratory determined the presence of infection by counting the number of parasites per 200 leukocytes, based on a mean count of 8,000 leukocytes per microliter of blood. Samples were negative when no parasites were detected in 200 high power (100x) fields. For the qPCR, DNA was extracted from a 6 mm^2^ fragment of placenta using the Chelex method described by Plowe *et al.*[25]. A qPCR was performed as described previously [26]. Briefly, samples were first tested for *Plasmodium* using a genus-specific set of primers and probe. The reaction was performed in a final volume of 25 μl containing 5 μl of DNA, 12.5 μl of TaqMan universal master mix (Applied Biosystems), 200 nM of each primer (Plasmo1 and Plasmo2), 50 nM of Plasprobe on the ABI 7500 FAST platform, under universal cycling conditions as published. Samples with a cycle threshold (Ct) value under 45 were tested in a duplex species-specific real-time PCR reaction for *P. falciparum* and *P. vivax*[26].

### Expression analysis of cytokines and markers of tissue damage and inflammation

Cytokines were measured in mRNA isolated from maternal whole blood and placental tissue, while markers of tissue damage, hypoxia and inflammation were measured in mRNA isolated from placental tissue. Relative quantitation for expression analysis was performed using a reverse-transcription real time PCR assay (RT-PCR). Total RNA was extracted using QIAamp RNA Blood Mini® (QIAGEN) and cDNA was synthesized using First Strand cDNA Synthesis® (Fermentas). The reaction was set up in a Roche LightCycler® or ABI 7500. Table 1 details the primers and probes used. The following genes were analysed for expression: Fas, FasL; COX-1, COX-2, HIF, VEGF, and the cytokines IL- 2, IL-4, IL-10, IFN-γ and TNF. The efficiency of the PCR reactions was determined based on mRNA extracted from a stimulated BeWo cell culture or peripheral mononuclear cells from a donor. Complementary DNA was serially diluted and expression of β-actin was used to normalize the assays using the delta delta CT method (ΔΔCt) as described previously [27].

**Table 1:** Primers and probes used in this study

**Marker and cytokine gene**	**Sequence (5′-3′)**	**Product Size (pb)**	**ID**
B-actin	F: CGAGCGCGGCTACAGCTT	58 pb	NM_001101
	R: CCTTAATGTCACGCACGATT		
	P: ACCACCACGGCCGAGCGG		
FasL	F: CTGGGGATGTTTCAGCTCTTC	231	NM_152872
	R: GTCCTGCTTTCTGGAGTGAAG		
Fas	F: AAGGAGTACACAGACAAAGCCC	170	NM_000043.3
	R: GGGTGGCTTTGTCTTCTTCTT		
COX1	F:AGCAGCTTTTCCAGACGACC	157 pb	NM_000962
	R:CGGTTGCGGTATTGGAACTG		
	P: CTGGCCTCAGCACTCTGGAATGACAA		
COX2	F: CCTGATCCCCAGGGCTCAAAC	79 pb	NM_000963
	R: TTGGTGAAAGCTGGCCCTCG		
	P: TGCCCAGCACTTCACGCATCAGTT		
VEGF	F: TCTACCTCCACCATGCCAAGT	102 pb	AB021221
	R: TGCGCTGATAGACATCCATGA		
	P: CCAGGCTGCACCCATGGCAGA		
HIF	F: CCAAATCCAGAGTCACTGGAACTT	96 pb	NM_001530
	R: AGGTGAACTTTGTCTAGTGCTTCCAT		
	P: TACCATGCCCCAGATTCAGGATCAGACAC		
IL-2	F: TGATTTTGAATGGAATTAATAATTACAAG	95 pb	NM_000586.3
	R: TTTCAGTTCTGTGGCCTTCTT		
	P: CCCAAACTCACCAGGATGCTCACATT		
IL-4	F: GCCTCACAGAGCAGAAGACTC	75 pb	NM_172348.1
	R: CAGTTGTGTTCTTGGAGGCA		
	P: TGCACCGAGTTGACCGTAACAGACA		
IL-10	F: CCTGGAGGAGGTGATGCCCCA	131 pb	NM_000572.2
	R: CAGCGCCGTAGCCTCAGCC		
	P: CAAGGCGCATGTGAACTCCCTG		
IFNγ	F: GAAGAATTGGAAAGAGGAGAGTGA	218 pb	NM_029419.12
	R: TGGACATTCAAGTCAGTTACCG		
	P: TTCCTTGATGGTCTCCACACTCTTTTGG		
TNF	F. GCCCAGGCAGTCAGATCA	74 pb	NM_000594.2
	R: GCTTGAGGGTTTGCTACAACA		
	P: CCCGAGTGACAAGCCTGTAGCCC		

### Quantification of apoptosis

Apoptosis in placentas was assessed by a TUNEL assay as described earlier [28]. Placental sections were deparaffinized and placed on Fisherbrand Superfrost Plus® slides. Detection of cells undergoing apoptosis was performed using the kit DeadEnd Colorimetric System® Promega TUNEL, according to the manufacturer’s protocol. The apoptotic index was calculated based on the proportion of TUNEL stained cells observed in 10 fields (40x). The test was performed in a blinded fashion, for this samples were assigned a code which was revealed once all results were available.

### Statistical analyses

Data were analysed using Excel and SPSS 19. Significance was set at p < 0.05. Non-parametric tests were used for data analysis and the U Mann–Whitney test and the Kruskal-Wallis test were used for correlations. According to the data distribution, the variable number of DNA copies/μl of template was grouped by convenience as follows: Group 1 (0 copies-no infection); Group 2 (0.01 to 1.99 copies/μl), Group 3 (2.00 to 100 copies/μl), and Group 4 (> 100 copies/μl) [16].

### Ethical considerations

Pregnant women or guardians (in case of <18 years of age) signed a voluntary consent form. The study involved a minor risk and approval was granted by the Comite de Etica of Instituto de Investigaciones Medicas, Facultad de Medicina, Universidad de Antioquia (Approval Certificate number IIM 889ADV).

## Results

All fifty placentas were negative for *Plasmodium spp.* by microscopy, and 25 of them were positive for infection by qPCR: 16 *P. falciparum*, six *P. vivax* and three mixed infection. The characteristics of the women from which placentas were collected are summarized in Table 2. In general, women were in their first or second pregnancy, most placentas resulted from full term gestations (≥38 weeks), haemoglobin levels were similar in the two groups and the mean birth weight was similar and within normal range (<2,500 grams) in all. Interestingly, 19/25 peripheral blood samples from mothers with positive placentas were also positive, either by microscopy or qPCR by the same *Plasmodium* species infecting the placenta.

**Table 2:** General characteristics of parturient women participating in the study, according to placental infection status by qPCR

**Characteristic**	**Placental malaria by qPCR**	
	No (N = 25)	Yes (N = 25)
Age of mother (years)	22.6 (15–32)	22.43 (range 14–36)
Number of gestations	1.84 (range 0–6)	1.91 (range 0–6)
Weeks of pregnancy	37.6 (range 32–41)	38.3 (range 27–41)
Mother’s haemoglobin (g/dL) at delivery	11.38 (range 7.15–18.30)	10.83 (range 9.00–13.30)
Identification of infecting species in mother’s peripheral blood	-	19* (11 *P. falciparum*, 7 *P. vivax*, 1 mixed)
Birth weight (grams)	2,891 (range 1,780–3,900)	3,305 (range 2,600–4,400)

*6 subjects were negative for infection in peripheral blood.

### Cytokine expression in pregnant women and placentas

Maternal peripheral blood and placental tissue were evaluated for the expression (mRNA) of IFN-γ, TNF, IL-2, IL-4 and IL-10. In both compartments, expression of IFN-γ, TNF, and IL-10 was significantly higher in PM + when compared to PM-. Meanwhile, expression of IL-4 was high in placentas in PM+, and IL-2 was high in peripheral blood of the same group (Table 3). In peripheral blood of the PM + group, the cytokines TNF and IFN-γ were elevated and positively correlated (rho = 0.749, P = 0.000 with n = 50). In addition, for IL-4 and IL-10 a lower (rho = 0.431, P = 0.002 with n = 50), but nevertheless significant, correlation was observed in placenta, but not in peripheral blood (rho = 0.048, P = 0.740 with n = 50). Other positive correlations included IL-2 and TNF (rho = 0.676, p = 0.000 n = 50), IL-2 and IFN-γ (rho = 0.760, p = 0.000 n = 50), all in peripheral blood. In this same compartment, IL-4 did not correlate (p ≤ 0.406), but IL-10 positively correlated with IL-2, TNF and IFN-γ (p = 0.000).

**Table 3:** Cytokine expression (Mean ratio ± standard deviation) in placental tissue and maternal peripheral blood according to the infection status

**Placental tissue**	**Infected**	**Uninfected**	**P (U Mann–Whitney)**
IL-2	1.00 ± 0.42	0.96 ± 0.30	>0.05
IFN-γ	9.00 ± 0.811	0.46 ± 0.37	0.0001
TNF	6.16 ± 0.75	0.83 ± 0.45	0.0001
IL-4	1.60 ± 0.40	1.05 ± 0.62	0.0001
IL-10	3.50 ± 0.93	0.18 ± 0.18	0.0001
**Maternal peripheral blood**			
IL-2	3.65 ± 1.11	1.18 ± 0.50	0.0001
IFN-γ	6.53 ± 2.38	1.10 ± 0.72	0.0001
TNF	1.53 ± 0.41	0.76 ± 0.46	0.0001
IL-4	1.63 ± 0.34	1.41 ± 0.50	>0.05
IL-10	2.45 ± 0.64	0.65 ± 0.50	0.0001

In general, the differences observed in expression of cytokines in peripheral blood and placenta between PM + and PM-, persisted when analysis was performed taking into account the infecting species (Table 4).

**Table 4:** Cytokine mean (standard deviation) expression ratio in placenta and maternal peripheral blood according to plasmodial species

**Cytokine**		**Placental tissue**		**Maternal peripheral blood**	
	**Group**^**a**^	**Mean (S.D.)**	P*	**Mean (S.D.)**	P*
**IL2**	PM-	0.95 (0.28)		1.18 (0.50)	
	PM + Pf	1.08 (0.43)		3.51 (1.20)	
	PM + Pv	0.95 (0.42)		3.92 (1.12)	
	PM + mixed	0.67 (0.29)	0.332	3.78 (0.70)	0.000
**IFNγ**	PM-	0.46 (0.37)		1.08 (0.72)	
	PM + Pf	9.00 (0.92)		6.06 (2.83)	
	PM + Pv	8.59 (0.57)		7.60 (0.78)	
	PM + mixed	9.33 (0.31)	0.000	6.85 (0.74)	0.000
**TNF**	PM-	0.83 (0.45)		0.76 (0.46)	
	PM + Pf	6.23 (0.91)		1.47 (0.43)	
	PM + Pv	6.05 (0.41)		1.48 (0.21)	
	PM + mixed	6.00 (0.00)	0.000	1.93 (0.46)	0.000
**IL4**	PM-	1.05 (0.62)		1.41 (0.50)	
	PM + Pf	1.56 (0.43)		1.64 (0.30)	
	PM + Pv	1.48 (0.21)		1.63 (0.49)	
	PM + mixed	1.96 (0.41)	0.024	1.52 (0.28)	0.634
**IL10**	PM-	0.18 (0.18)		0.65 (0.50)	
	PM + Pf	3.33 (1.04)		2.23 (0.54)	
	PM + Pv	3.57 (0.69)		2.93 (0.69)	
	PM + mixed	4.08 (0.61)	0.000	2.68 (0.58)	0.000

^**a**^PM- without placental malaria (n = 25); PM + Pf with placental infection by *P. falciparum* (n = 16)*;* PM + Pv with placental infection by *P. vivax* (n = 6)*;* PM + mixed with placental by both species (n = 3).

*(Kruskal-Wallis).

### Apoptosis, inflammation and hypoxia markers in placental malaria

According to TUNEL assay, the mean apoptotic index observed in the group PM + was 62.37% (range 55-79%) (Table 5), and this was significantly higher when compared with the PM- group (44.88%). Similarly, mean Fas expression was significantly higher in PM + than in PM- (6.52 *vs*. 3.42). However, FasL expression was higher in PM- than in PM + (6.95 *vs*. 5.60, p < 0.05) (Table 5).

**Table 5:** Ratio of expression and proportion of apoptotic cells in placental tissue in infected and uninfected placentas

**Marker**	**Infected (n = 25)**	**Uninfected (n = 25)**	**P (U Mann–Whitney)**
Apoptosis			
Apoptotic cells	62.37% ± 10.02%	44.88% ± 2.15	0.0001
Fas	6.53 ± 2.49	3.42 ± 0.84	0.0001
FasL	5.60 ± 2.13	6.95 ± 1,16	0.003
Hypoxia			
HIF	0.96 ± 1.15	0.21 ± 0.22	0.0001
VEGF	0,14 ± 0.14	0.12 ± 0.15	>0.05
Inflammation			
COX-1	6.95 ± 3.14	0.73 ± 0.41	0.0001
COX-2	2.86 ± 0.99	0.42 ± 0.29	0.0001

The inflammation markers HIF and VEGF were observed high in the PM + group, but only VEGF was significantly different. As for the hypoxia markers, COX-1 and COX-2, both were significantly high in the PM + group. In general, all evaluated markers of tissue damage were up regulated in the placentas of the PM + group (Table 5).

Analysis of apoptosis, inflammation and hypoxia markers according to the *Plasmodium* species observed in placenta, confirmed similar results to those observed between the PM + group and non-infected placentas (Table 6).

**Table 6:** Mean value (standard deviation) of Apoptosis index and ratio (standard deviation) of expression of inflammation and hypoxia markers according to plasmodial species infecting the placenta

	**PM-**^**a**^	**PM + Pf **^**a**^	**PM + Pv **^**a**^	**PM + mixed**	P*
**Apoptotic cells index**	44.78 (2.15)	71.32 (4.45)	61.04 (5.96)	57.56 (3.06)	0.000
**Fas**	3.2 (0.84)	5.18 (2.11)	6.57 (1.61)	10.49 (2.09)	0.000
**FasL**	6.74 (1.16)	4.49 (2.47)	5.5 (2.82)	5.68 (2.3)	0.009
**HIF**	0.1 (0.22)	0.37 (0.66)	0.35 (0.47)	0.82 (2.97)	0.000
**VEGF**	0.05 (0.15)	0.09 (0.17)	0.06 (0.07)	0.09 (0.06)	0.412
**COX1**	0.43 (0.41)	6.64 (3.54)	5.22 (0.69)	5.9 (3.4)	0.000
**COX2**	0.24 (0.29)	2.45 (0.93)	2.71 (0.82)	2.19 (1.82)	0.000

^**a**^PM- without placental malaria (n = 25); PM + Pf with placental infection by *P. falciparum* (n = 16)*;* PM + Pv with placental infection by *P. vivax* (n = 6)*;* PM + mixed with placental infection by both species (n = 3).

*(Kruskal-Wallis).

### Relationship between markers of apoptosis/inflammation/hypoxia and cytokine expression in placenta

No significant correlation was observed between IL-2 and any of the apoptosis/inflammation/hypoxia markers. Similarly, VEGF failed to correlate with the expression of any cytokine. On the other hand, the percentage of apoptotic cells and the expression of Fas, COX1, COX2 and HIF positively correlated with IFN-γ, TNF, IL-4 and IL-10. Meanwhile, FasL negatively correlated with the remaining variables (Table 7).

**Table 7:** Correlation (rho, p) between the expression of cytokines and the expression of markers of apoptosis, hypoxia and inflammation in placental tissue, regardless of the infection status (n = 50)

	**INFγ**	**TNF**	**IL-4**	**IL-10**
**Apoptotic cells (%)**	0.709 (0.000)	0.708 (0.000)	0.398 (0.004)	0.778 (0.000)
**Fas**	0.673 (0.000)	0.646 (0.000)	0.370	0.659 (0.000)
**FasL**	−0.385 (0.006)	−0.281 (0.048)	−0.343 (0.015)	−0.437 (0.002)
**COX1**	0.794 (0.000)	0.857 (0.000)	0.457 (0.001)	0.763 (0.000)
**COX2**	0.784 (0.000)	0.826 (0.000)	0.307 (0.030)	0.784 (0.000)
**HIF**	0.701 (0.000)	0.570 (0.000)	0.249 (0.081)	0.565 (0.000)

### Relatioship between biomarkers of apoptosis/inflammation/hypoxia and placental parasite density in placenta

Placental parasite density was calculated using a qPCR assay as detailed in the methods section. In order to facilitate analysis, and based on the frequency distribution of data, results were categorized according to the copies of parasite DNA measured per PCR μl of total purified DNA: Group 1 (0 copies-no infection); Group 2 (0.01 to 1.99 copies/μl), Group 3 (2.00 to 100 copies/μl), and Group 4 (> 100 copies/μl) [16]. In general, significant differences were observed between Group 1 and the remaining groups as a whole. Copy number correlated positively and significantly with the percentage of apoptotic cells, and expression of Fas, COX1, COX2, HIF, IFN-γ, TNF, IL-4 and IL-10 (p = 0.000 and for IL4 p = 0.017). However, no significant correlation (p ≥ 0.078) was observed between expression of FasL, VEGF and IL-2 in relation to the copies of parasite DNA.

## Discussion

Although the effects of malaria infection on the mother and the foetus depend upon multiple factors, the host immune response and the histopathology of the placenta are specifically associated with complications observed before birth and around the neonatal period [3]. This study evaluated the important immunological aspects and molecular mechanisms associated with tissue damage in the placenta, which contribute to the understanding of the pathophysiology of placental infection. Complex cellular and molecular mechanisms, such as apoptosis, hypoxia and inflammation have not been evaluated in cases of placental infection by *Plasmodium spp* in this endemic region*.* Such mechanisms are important to a healthy pregnancy since they contribute to implantation and promote placental development [16].

This is a first exploratory study on the subject in women from a region where both *P. falciparum* and *P. vivax* are endemic and is the first study to report on apoptosis and tissue injury during submicroscopic placental infection. All studied groups came from the same malaria endemic region as to control for any socio-epidemiology associated bias. According to a recent report [16], most placental infections at delivery in this region were sub-clinical and thick smear microscopy examination failed to diagnose them. Based on these findings, the current study included subjects in which diagnosis of infection was confirmed by qPCR.

Changes in apoptosis have been scarcely studied in placental malaria. In other pathologies such as preeclampsia, the high rate of apoptosis has been directly linked with intrauterine growth restriction [29]. Importantly, the apoptotic index in the PM + placentas herein studied was higher than in PM-. Early reports on apoptosis in malaria infected placentas showed no difference in the proportion of apoptotic cells when compared to uninfected ones [30]. A possible explanation for the dissimilar results from those reported here might be the application of different techniques- TUNEL *vs.* haematoxylin-eosin staining.

Fas and FasL expression have been proposed as markers of apoptosis, and this is considered an important mechanism by which cytokines act locally and may influence critical signaling processes. In *in vitro* studies with cell models and placental tissue explants stimulated with pro-inflammatory cytokines, the expression of Fas and FasL was high and similar to the results reported here [31]. Taken together, these observations implicate apoptosis as a host response or a downstream effect of malaria infection in the placenta.

In the current study, increased apoptosis in infected placentas was also associated with high expression of the pro-inflammatory cytokines IFN-γ and TNF. Similar observations with increased expression of Th1 cytokines such as IL-2, TNF, IFN-γ and decreased Th2 cytokines such as IL-6 and TGF-β have been reported by others [31], and haemozoin deposition and haemozoin-loaded macrophages have implicated in the induction of inflammation in the throphoblast [30,32]. Ongoing studies are exploring the association between haemozoin deposits and the cytokine profile in infected placentas in Colombia.

Other authors, based on high levels detected in serum of women with gestational malaria, have proposed IL-10 as biomarker of placental infection [33] and a definitive marker of inflammation during placental malaria [34]. This hypothesis is supported in the current study by the higher expression of IL-10 observed in the infected *vs.* uninfected placentas. The increased expression of this cytokine in cases of submicroscopic infection is a novel and promising finding.

Hypoxia has been associated with complications from placental malaria infection [35]. However, few studies have explored the relationship between malaria and placental hypoxia [36]. Common histological changes associated with infection such as basal membrane thickening, mononuclear infiltrates and presence of parasites in the intervillous space, affect oxygen transport across the placenta. Some hypoxia mechanisms include oxygen consumption by infiltrating cells, decreased blood perfusion and reduction of the effective foetal-maternal surface area [36]. In consequence, a physiological adaptation to hypoxia results in syncytial knot formation, an alteration often reported during placental infection by *Plasmodium*[8]. Similar to the current report, Boeuf and colleagues in 2008, observed increased expression of HIF-1α and VEGF during placental infection, however not direct association between placental malaria and hypoxia could be confirmed [12].

Cyclooxygenase (COX) and lipoxygenase (LOX) transform fatty acids into prostaglandins and leukotrienes, which play important roles in pregnancy and foetal development. In addition, COX-2 has been proposed as a marker of preeclampsia and recovery after infectious conditions [37]. In a study published by Sarr *et al*., increased expression of COX-2 and IL-10 in chronic placental malaria was detected, and COX-2 was associated with maternal anaemia, placental macrophage infiltration and haemozoin deposition [13]. These results are consistent with the findings herein reported regarding increased COX-2 and IL-10 in cases of placental infection. Furthermore, COX-1 is reported for the first time in association with placental malaria.

Interestingly, no differences were observed between the changes in the markers studied in *P. vivax* infected placentas compared with those infected with *P. falciparum*. Some complications of *P. vivax* infection might be explained by the production of pro-inflammatory cytokines, which alter the balance at the foetal-maternal interface. Based on this, it is hypothesized that *P. vivax* is as pathogenic as *P. falciparum*, regardless of the presence of sequestering parasites.

The knowledge gathered so far on the pathophysiology of placental malaria mainly describes cases of *P. falciparum* infection. The current results demonstrate that *P. vivax* might also be equivalent in the pathophysiology of placental damage. These results need to be complemented with a study of a large series of *P. vivax* infected placentas. In addition, the role of naturally acquired specific humoral immunity should complement these studies in order to clarify its implication on clinical status. Finally, studies of the consequences of *P. vivax* placental infection in Asia and America in the context of a pregnancy vaccine and intermittent preventive therapy should be a priority.

In conclusion, submicroscopic placental infection by malaria parasites failed to induce major clinical effects on mother or foetus but associated with a pro-inflammatory cytokine profile, regardless of the infecting species, and this was more evident in the placental tissue. Finally, increased apoptosis was common in *Plasmodium spp*. infected placentas.

## Competing interests

The authors affirm that they have no commercial or other association that might pose a conflict of interest.

## Authors’ contributions

OA, EA BA designed and performed the analysis in blood samples. OA, JC-F and AM conceived the project and designed the experiments, supervised overall design and development and wrote the manuscript. SKY designed the experiments. All authors read and approved the final manuscript.

## Financing

This work was supported by the Colciencias (project codes 111549326134 and 111556933361); the Universidad de Antioquia (project code IIM 8764–2557); Estrategia de Sostenibilidad 2013–2014, and Banco de la Republica (code 3069).
